# Liquid-like behaviours of metallic glassy nanoparticles at room temperature

**DOI:** 10.1038/s41467-019-09895-3

**Published:** 2019-04-29

**Authors:** C. R. Cao, K. Q. Huang, J. A. Shi, D. N. Zheng, W. H. Wang, L. Gu, H. Y. Bai

**Affiliations:** 1 Institute of Physics, Chinese Academy of Sciences, Beijing, 100190 P. R. China; 2 Center of Materials Science and Optoelectronics Engineering, University of Chinese Academy of Sciences, Beijing, 100049 China; 3 Songshan Lake Materials Laboratory, Dongguan, Guangdong, 523808 China

**Keywords:** Nanoparticles, Nanoscience and technology

## Abstract

Direct atomic-scale observations and measurements on dynamics of amorphous metallic nanoparticles (a-NPs) are challenging owing to the insufficient consciousness to their striking characterizations and the difficulties in technological approaches. In this study, we observe coalescence process of the a-NPs at atomic scale. We measure the viscosity of the a-NPs through the particles coalescence by in situ method. We find that the a-NPs have fast dynamics, and the viscosity of the a-NPs exhibits a power law relationship with size of the a-NPs. The a-NPs with sizes smaller than 3 nm are in a supercooled liquid state and exhibit liquid-like behaviours with a decreased viscosity by four orders of magnitude lower than that of bulk glasses. These results reveal the intrinsic flow characteristics of glasses in low demension, and pave a way to understand the liquid-like behaviours of low dimension glass, and are also of key interest to develop size-controlled nanodevices.

## Abstract (web summary)

Nanoscale materials often exhibit size-dependent behaviour. Here, the authors use electron microscopy to quantitatively study the size-related dynamics of amorphous metallic nanoparticles, finding that particles below a critical size are in a supercooled liquid state at room temperature, with a viscosity much lower than that of bulk glasses.

## Introduction

Myriad applications in modern science and technology including nanoscale mechanics, magnetic, electronics, optics, catalysis and nanomedicine, are all related to the size effects of materials^1,2^. Recently, accompanying with the development of nanotechnology, abundant outstanding performances, properties and features have been discovered in nano-scale materials^3,4^. However, a great number of studies focus on the nano-crystalline materials, the nature of low-dimensional dynamics of amorphous materials is hitherto poorly understood. Therefore, a deep comprehension of dynamics of low-dimensional amorphous materials is a frontier research which is still in its infancy. The research concerns about how the dynamics and properties of amorphous materials will change with the decrease of their sizes and how these changes reflect the intrinsic features of the materials^5–7^. In addition, in the sense of basic research, the precisely quantitative information about dynamics is remarkably essential for the stability of sub-10 nm materials at room temperature in practical applications.

The atomic metallic glass with a structure close to the dense random packing hard spheres is a model system for studying fundamental issues in materials. Recently, it is found that the nano-sized surface layer of glasses has a pronounced higher mobility than that in bulks^8–10^, and the fast surface dynamics induces surface crystallization^11^, ultrastable glass formation^12,13^ and some special mechanical behaviors in the nanoscale^14,15^. Glassy materials do exhibit different physical and chemical properties from that of bulks at nanoscale. However, how the dynamics of amorphous materials will change in the miniaturization process down to nanoscale is still elusive due to the fact that direct atomic scale observation and measure of dynamics for low-dimensional amorphous metallic materials are very difficult and continuously challenging.

Viscosity is one of the most important dynamic properties of amorphous material, and has been hitherto measured sparingly in nano-scale owing to the technology difficulties and less consciousness to its striking difference between bulk and low dimensional forms^16,17^. In this work, taking advantages of the atomic high-resolution transmission electron microscopy (HRTEM) and the atomic resolution aberration-corrected scanning transmission electron microscopy (Cs-STEM), we show the measurements of size dependent viscosity of the amorphous metallic PdSi nanoparticles (NPs). Based on the classical coalescence models of NPs^18–21^, the viscosity of the amorphous NPs (a-NPs) is quantitatively estimated through the electron beam-induced coalescence process. The results provide the direct and quantitative evidences of the fast dynamic characterization of a-NPs, and this might lift the veil on the glass transition and intrinsic flow feature of glasses and is also beneficial for developing new size-controlled nanodevices.

## Results

In situ observations of coalescence process under HRTEM. For in situ dynamic measurement of metallic NPs, the image drifting is a major difficulty to be overcome. The drifting could be eliminated as much as possible if the experiments are performed at room temperature (RT). In addition, under the conditions of electron beam irradiation the pure metallic NPs can easily react with siliceous substrates. After intensive tests we found that the PdSi a-NPs have not only the outstanding chemical stability, but also an observable mobility at RT in the nano-scale. Therefore the PdSi alloy was chosen as a model system. The Pd_80_Si_20_ NPs were deposited via pulsed laser deposition (PLD) in a high vacuum (10^−5^ Pa) chamber at RT. The electron transparent TEM window grids were used as the substrates (from SiMPore, Inc.), which were supported with amorphous silicon nitride films. The congruence of composition from target to film in PLD was stable over a wide range of composition^22^. Since Pd_80_Si_20_ has excellent glass forming ability^23^, we used a glassy Pd_80_Si_20_ PLD target to ensure the film being formed with a-NPs. Before deposition we heated the substrates at 300 °C for 15 min in a high vacuum to desorb volatile contaminants on the surfaces. After cooling the substrates to RT, a 2 Hz KrF laser (wavelength: 248 nm) with about 300 mJ cm^−2^ incident laser fluence was employed for depositing the ultrathin films (thickness < 10 nm). The higher surface energy of Pd (1.8 J m^−2^)^24^ and relatively low interface energy between the PdSi a-NPs and silicide substrate (<0.6 J m^−2^)^25^ led to the formation of the three-dimensional islands. The fresh samples were transferred into TEM within 10 min to avoid the structure relaxation and pollution. The coalescence processes of the NPs were detected by the HRTEM and the Cs-STEM (JEOL-ARM200F with cold field-emission gun (CFEG)).

**Fig. 1:**
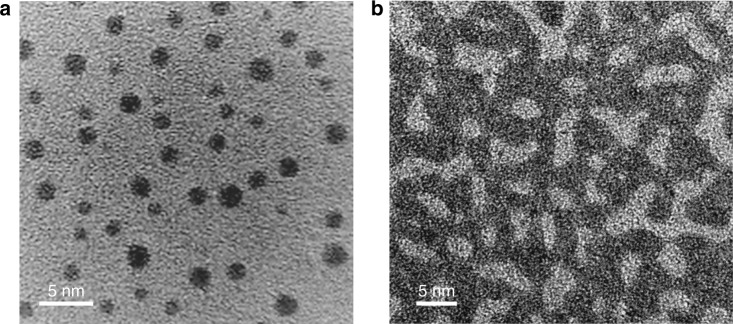
Bright filed HRTEM images of the PdSi ultrathin films. **a** The discrete amorphous PdSi nanoparticles (NPs) deposited with low dose (one dose) with size range of 1 ~ 3 nm. **b** The thin PdSi film deposited with high doses (five doses) shows the bicontinuous pattern

Figure 1 shows the bright filed HRTEM images of the PdSi films deposited with different doses. An ultrathin film deposited with a low dose (denoted as one dose) exhibits the discrete PdSi nanoparticles (NPs) with size range of 1 ~ 3 nm (Fig. 1a). As shown in Fig. 1a, the NPs with sizes of 1 to 3 nm have the round edges without any lattice fringe, indicating most particles are in amorphous state. Figure 1b shows the film deposited with higher doses (about five doses compared with the low dose ultrathin film), and it can be seen that the spherical particles have connected to each other and formed the continuous regions with bicontinuous patterns with the width ranging from 3 to 6 nm.

**Fig. 2:**
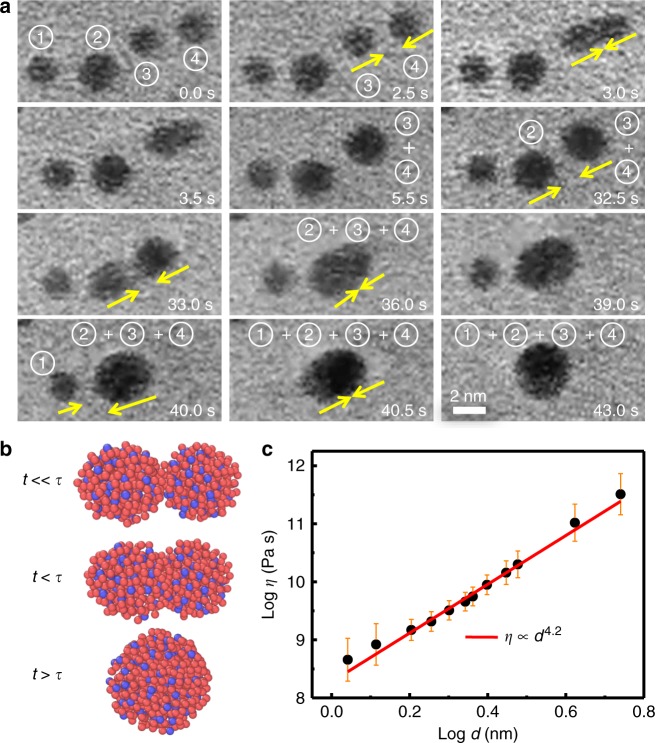
Liquid-like behaviours of metallic glassy nanoparticles. **a** A series of images from Supplementary Movie 1 show the coalescence of the four NPs marked as ➀, ➁, ➂, ➃. They merged into round spheres within 40 s, and each image shows the distinguish step. The diameters of the NPs ➀, ➁, ➂, ➃, ➂ + ➃,➁ + ➂ + ➃,➀ + ➁ + ➂ + ➃ are 1.8, 2.2, 1.9, 1.9, 2.3, 2.8, and 3.0 nm, respectively. **b** Schematics shows the relation between the observation time *t*, coalescence relaxation time *τ* and degree of coalescence. **c** A power law relationship between the viscosity *η* and the diameter *d* of a-NPs. The error bars show the standard deviation from 3 to 6 repeats of the measurements

The HRTEM observation of the low dose film shows that there were no obvious crystalline nucleus in the initial state of the film. When the film was irradiated by the detecting electron beam with energy comparable to the binding energy between the NPs and the substrate^26^, the NPs start to move instantly and randomly on the substrate, similar to the Brownian-like motion in the local areas (see Supplementary Movie 1). It has been observed that the NPs grew in the ways of Ostwald ripening and frequent coalescence events^27^. Owing to higher atomic mobility in amorphous alloy^10^ and lower restriction from the substrate^28,29^, it is allowed to observe the features of coalescence process directly. Figure 2 shows the liquid-like behaviours of metallic glassy nanoparticles. A series of bright-field TEM images (Fig. 2a) were extracted from the Supplementary Movie 1, which exhibits the coalescence process of the a-NPs under a 200 kV, 2.1 × 10^6^ electrons nm^−2^ s^−1^ electron beam irradiation and the movie images were taken with intervals of 0.5 s per frame. The a-NPs were activated when they were irradiated, and minimized their surface energy through reducing surface areas. Almost all the NPs completed the coalescence events in several minutes. As an example, Fig. 2a shows the coalescence processes of the a-NPs marked as ➀, ➁, ➂ and ➃ with the sizes ranging from 1.6 nm to 2.3 nm. The coalescence process has two steps: first, the a-NPs approach each other driven by random thermal motion of the NPs and surface atomic traction forces between NPs^30^, then the NPs touch each other and merge to form a spherical particle. The a-NPs ➂ and ➃ approached each other between 2.5 s and 3.0 s, and the coalescence finished at 5.5 s. The merged NP ➂ + ➃ started to approach to the NP ➁ at 32.5 s, contacted each other before 33.0 s, and decreased their center-to-center distance to minimize the surface area and finished the coalescence at 40.0 s. During contacting a transient neck-like structure was formed by aligning planes of contacting surface, which induced a larger atomic potential gradient and yielded faster evolution^20,31,32^. Consequently, they merged and formed a spherical particle gradually within 7.5 s, and the whole coalescence finished at 40.0 s. Subsequently, the NPs ➀ and ➁ **+** ➂ **+** ➃ approached to each other from 40.0 s to 40.5 s, formed a larger spherical particle and finished their coalescence at 43.0 s, and all three coalescence processes of the four NPs finished within 43.0 s.

### Estimation of the viscosity by the coalescence processes of a-NPs

The above observation results demonstrate that the coalescence of the a-NPs with different sizes takes different times. Based on the Frenkel model^18^, when the a-NPs in a fusion pair have similar sizes, the dynamics of a-NPs can be estimated from the coalescence relaxation time, *τ*, defined as the time period from the touch of two particles to the formation of a larger and complete spherical particle as: *τ* = *ηd*/*γ*, where *η* is the viscosity, *d* is the diameter of the smaller particle in the fusion pair in its initial state, and *γ* is the surface tension. Figure 2b depicts the degree of coalescence at a given time using parameter *τ*. When the observation time *t* is longer than the time *τ*, the coalesced particle can acquire a complete spherical shape. We selected smaller NPs so that their entire coalescence processes can be observed in the limited observation time. From touching to forming a spherical shape of NPs, it takes different times for NPs with different sizes. For example, the relaxation time *τ* of the coalescence of the NPs ➂ and ➃ is 3 s, and the NPs ➂ and ➃ have the same diameter of 1.9 nm. The surface tension of Pd-based bulk metallic glass is reported to be about 2.0 ± 0.2 J m^−2^ ref. ^10,24^. Considering the size effect, the surface tension of NPs could be presented as *γ*(*d*) / *γ*_0_ = 1/(1 + 4*δ*/*d*), where *γ*_0_ is the bulk value, and *δ* is Tolman’s length which is taken as a constant for a kind of metal, and it approximately equals to the value of bond length (for Pd *δ* ≈ 0.2751 nm)^33–35^. From *τ* = *ηd*/*γ*, the viscosity of the NP ➂ in the fusion pair ➂ and ➃ was estimated to be on the order of magnitude of 10^9±1^ Pa·s. For the fusion of particles ➁ (2.2 nm) and ➂ + ➃(2.3 nm), the longer relaxation time of 7.5 s was required, and the viscosity of particle ➁ is estimated to be about 10^9±1^Pa·s. The viscosity is four orders of magnitude less than that of glasses (10^13^ Pa·s) at the glass transition temperature *T*_g_, indicating that all these NPs were in a supercooled liquid state at RT^36–38^.

### The size dependence of the dynamics of a-NPs

To further investigate the influence of size on the dynamics, we observed the coalescence processes under the same probing conditions in the film deposited with high doses (see Fig. 1b, Supplementary Movie 2 and Supplementary Figure 1). The coalescence of the initial bicontinuous particles moved so sluggishly that we could not obtain the entire process in the observable timescale of 10^3^ s. The viscosity was estimated to be *η* ≥ 10^11^ Pa s, which is more than two orders of magnitude higher than that of 2 nm NP, suggesting the mobility of atoms in NPs decreases with increasing size of NPs. We estimated *η* of the smaller NP in the fusion pairs with various sizes, and the *η* (or *τ*) and *d* exhibit a power law relationships: *η* ∝ *d*^ 4.2^ (or *τ* ∝ *d* ^5.2^) as shown in Fig. 2c, indicating the dynamics of the a-NPs is very sensitive to their sizes. The constraint from the particle-substrate interfacial adhesion is weak^39^ compared with the interaction between NPs^40^.

The coalescence for the crystalline NPs (c-NPs) can be predicted by the classical model as:^19,20^

1 $$\tau \ast = \left( {k_{\mathrm{B}}T/CD_s\gamma } \right)\left( {d/a} \right)^4,$$

where τ* is the coalescence time of this model, *C* is the numerical constant, *D*_s_ is the surface diffusion coefficient, and *a* is the atomic size. At a constant temperature *D*_s_ can be regarded as a constant. In the formula *τ* = *ηd*/*γ* for an amorphous coalescence a bulk value *η* is involved, and in the formula τ* = (*k*_B_*T*/*CD*_s_*γ*)(*d*/*a*)^4^ for a crystal only surface diffusion *D*_s_ is included due to the fact that in a crystal only surface atoms and atoms in grain boundaries behavior in the similar form as that in amorphous coalescence. Actually, *τ* = *ηd*/*γ* and τ* = (*k*_B_*T*/*CD*_s_*γ*)(*d*/*a*)^4^ are similar, and both of them count atoms moving in the string-like cooperation form, and this enables us to compare the dynamics of amorphous and crystal coalescence by their relaxation time *τ* and τ*, respectively^41,42^. From Eq. (1) the τ* and *d* have a relation of τ* ∝ *d* ^4^, which is consisted well with other studies of crystalline Pd NPs^43,44^. Comparing the results *τ* ∝ *d* ^5.2^ for a-NPs and τ* ∝ *d* ^4^ for c-NPs, we find that the diffusion in a-NPs increases faster than that in c-NPs with decreasing size of NPs, implying that a-NPs have a more sensitive relationship with dimension than that of c-NPs.

**Fig. 3:**
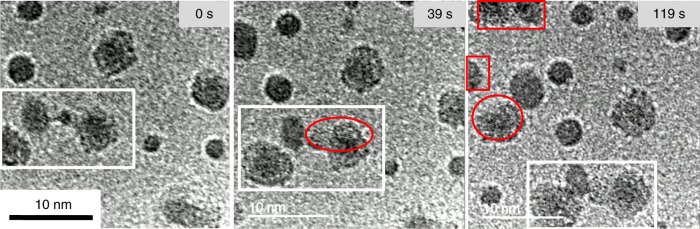
The coalescence process of the large NPs with the long time irradiation. A series of images from Supplementary Movie 4 shows the coalescence process of the large NPs which were exposed under the probe electron beam for 10 min. The large size NPs exhibit a rather slow merging rate as the NP group marked with white frame. The irradiation with the long time induced the observable crystalline lattice fringes in the areas marked with the red frames

The size effects of *η* ∝ *d* ^4.2^ or *τ* ∝ *d* ^5.2^ (equivalent relations derived from the formula *τ* = *ηd*/*γ*) for the a-NPs would break down when the sizes of a-NPs are too small or too large. The reasons are as follow: When the NP size is smaller than ~ 1.5 nm, the effect of substrate is evident and the ripening would be prior to the coalescence (see Supplementary Movie 3 and Supplementary Figure 2), i.e. the combination of the substrate effect and the ripening would make the relation of *η* ∝ *d *^4.2^ break down. In the other hand, when the size of NP is too large, both the possible crystallization and much lower fraction of the surface atoms would also make the relationship of *η* ∝ *d* ^4.2^ break down. To study the influence of the crystallization on the merging rate, the larger NPs with sizes of 2 ~ 6 nm were exposed under the probe electron beam for 10 min. Figure 3 shows the coalescence processes of the large NPs with the long time irradiation. As shown in Fig. 3 the long time irradiation induced the observable crystalline lattice fringes in the large NPs (*d* > 3 nm), and the c-NPs exhibit a rather slow merging rate (see Supplementary Movie 4).

### In situ observations of coalescence processes under Cs-STEM

**Fig. 4:**
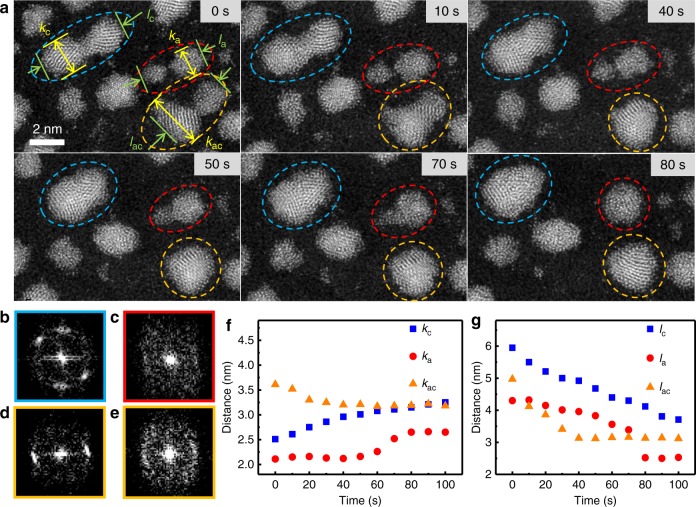
In situ observation of coalescence process under Cs-STEM. **a** A series of Cs-STEM images with the times as labeled. The coalescence of the c-NP, a-NP and a-c-NP pairs are marked by blue, red and orange dashed circles, respectively. **b** The local area fast Fourier transforms (FFT) of the c-NP pair (in blue circle) at 0 s. **c** FFT of the a-NP pair (in red circle) at 0 s. **d**, **e** FFT of the c-NP and a-NP of the a-c-NP pair (in orange circle) at 0 s, respectively. **f**, **g** The time dependences of the sizes of the NP pairs, marked as distances of *k*_c_, *k*_a_, *k*_ac_, *l*_c_, *l*_a_, and *l*_ac_, respectively. The surface liquid-like layer in the c-NPs accelerated and finished the coalescence process before rotating of the c-NPs to match perfect lattice

**Fig. 5:**
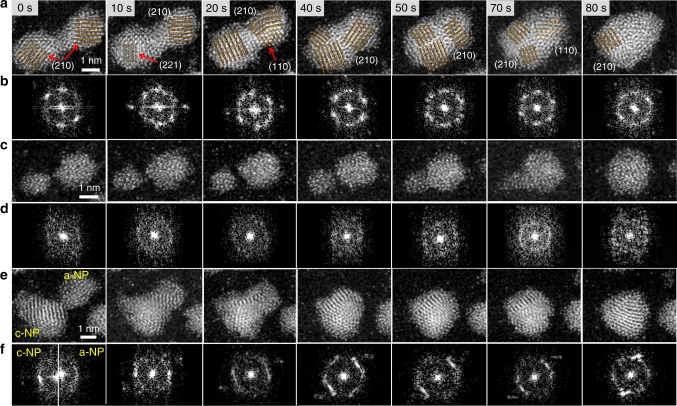
Details of evolution of the three coalescence processes upon the time. A series of Cs-STEM images with the irradiation times as labeled (Corresponding to the three NP pairs in Fig. 4). **a**, **c**, **e** The coalescence of the c-NP, a-NP and a-c-NP pairs, respectively. **b**, **d**, **f** The corresponding FFT of the c-NP, a-NP and a-c-NP pairs, respectively. To show the rotation of the c-NP pair, the crystal planes are marked in **a**. The separated two parts in **f** at 0 s refer to the FFT of the c-NP and a-NP in the a-c-NP pair, respectively

**Fig. 6:**
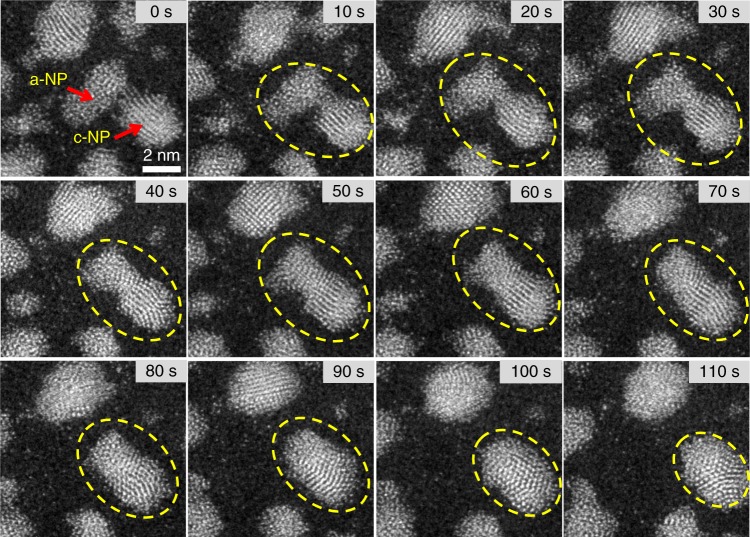
Cs-STEM images of a coalescence between the a-NP and c-NP. Within 100 s the a-NP approached, contacted and merged into the c-NP gradually

To further study the dynamic difference between the coalescence process of a-NPs and c-NPs at the same time, we prepared the thin film with both amorphous and crystalline PdSi NPs. When the diameter of the NPs is larger than 2.5 nm, the crystallization induced by irradiation would occur^45^. We thus applied the atomic resolution Cs-STEM to distinguish three different coalescence processes of a-NP, c-NP and amorphous crystalline NP (a-c-NP) pairs, and studied the crystallization influence on the NPs dynamics. We slowed down the merging rate of the NPs by applying a 200 kV cold field emission which was equivalent to the electronic current density ~ 0.5 × 10^6^ electrons nm^−^^2^ s^−1^. The time delay between the acquisitions of the two images was 2 s, and the exposure time was 8 s. Figures 4–6 show the details of the Cs-STEM images for the coalescence of a-NP, c-NP and a–c-NP pairs. We can distinguish the three coalescence processes through a series of Cs-STEM images and the corresponding local area fast Fourier transform (FFT) (Fig. 4a-e, Fig. 5). Figure 5 gives more details about the time evolution of the three coalescence processes. At 0 s, two c-NPs started to attach each other and formed a peanut shape NP, while the two a-NPs still remained untouched (Fig. 4a). Under the scanning electron radiation, the c-NP pair diffused continuously and the neck size increased until the two NPs formed an oval shape particle. Different from the continuous progressive coalescence of the c-NP pair, the *l*_a_ (the length along the center-to-center direction of the NP fusion pair) and *k*_a_ (the width vertical to *l*_a_) of the a-NP pair show the abrupt changes from 70 to 80 s and 60 to 70 s, respectively (Fig. 4f, g). The coalescence of the a-NPs involves hydrodynamic flow driven by the surface tension forces as liquid NPs^20^ without the processes of rotating or gliding to find low energy boundary or matching angle at the interface^46^. The two a-NPs touched at 50 s, and at 70 s the smaller one retarded its evolution into an acute triangular tail, which induced the jumps of *l*_a_ and *k*_a_ (*l*_a_ jumped between 70 and 80 s later than the jump of *k*_a_ between 60 and 70 s). The a-NP pair finished the coalescence within 30 s while the c-NP pair took more than 100 s. The a-c-NPs touched each other at 0 s, and the a-NP merged into the c-NP quickly within 10 s. Finally the a-c-NP pair formed a c-NP with a non-perfect spherical shape^32,47^ and completed the coalescence at about 40 s. The results show that a-NPs have higher mobility than that of c-NPs and the coalescence of the a-c-NP pair has a moderate mobility. Figure 6 shows the coalescence of another a-c-NP pair with similar particle sizes under the similar conditions. It could also be seen that the a-NP has much higher mobility than that of the c-NP. As shown in Fig. 6, the a-NP approached, contacted and merged into the c-NP gradually. During the coalescence the epitaxial lattice grew from the c-NP to a-NP. Referring to the surrounding particles the center of a-NP moved from the initial site to c-NP within 100 s, in contrast the shape and site of c-NP remained almost unchanged, indicating that the a-NP has evidently higher mobility than that of the c-NP.

Under this lower irradiation condition of Cs-STEM, we analyzed dozens of the a-NP pairs with the diameters ranging from 1 nm to 3 nm. The full transformation into a spherical shape leads to an estimated viscosity of 10^10^^± ^^1 ^Pa·s by the Frenkel model. It is only slightly higher than the value obtained under the HRTEM, indicating that the viscosity detected by different modes with different electron irradiation intensities have tolerable errors. The temperature rise induced by the electron beam irradiation in TEM in our experiments is smaller than 10 K^48–51^, which is far below the *T*_g_ of glassy Pd_80_Si_20_ (607 K).

## Discussion

The direct atomic-scale observations and quantitative measurements on dynamics of the amorphous NPs show that the low dimensional NPs are in a supercooled liquid state and exhibit liquid-like behavior when the sizes are smaller than about 3 nm. The a-NPs have fast dynamics, manifesting as the viscosity of the a-NP (*η* = 10^9±1^Pa·s) is four orders of magnitude lower than that of bulk glasses (*η* ≥ 10^13^ Pa·s) at room temperature, and the surface mobility is higher than that of their crystalline counterparts. Meanwhile, the viscosity and size of the a-NPs exhibit a power law relationship: *η* ∝ *d* ^4.2^ or *τ* ∝ *d* ^5.2^ (For comparison, τ* ∝ *d* ^4^ for the c-NPs), implying the diffusion in a-NPs increases faster than that in c-NPs with decreasing size of NPs, i.e., a-NPs have a more sensitive relationship with dimension compared to c-NPs.

The findings are helpful for understanding the nanoscale amorphization of monatomic metal^52^ and improved plasticity of metallic glasses in nanoscale^53^. With size decreasing, the structure of NPs is destabilized by the strong surface effect, and the atomic motion presents liquid-like behavior, which brings out the importance of the stability and mobility of NPs. It is also important for manipulating the properties of metallic liquids and glasses in nanoscale. Recent studies have shown the possibility of controlling the motion of nanomaterials^54^. The quantitative analysis of size effect on the viscosity, mobility and diffusion dynamics is of crucial importance for comprehension of supercooled liquid, dynamics of glass transition, and even the glass-forming ability in atomic scale. These liquid-like amorphous drops also can be developed into the shape-controlled metallic nanodevices with finite forces and much higher mobility^55^.

## Methods

### Sample preparation

The Pd_80_Si_20_ NPs were deposited via pulsed laser deposition (PLD) in a high vacuum (<10^-5^ Pa) chamber at RT. During the deposition the surface temperature of the substrate was lower than 100°C. The electron transparent TEM window grids were used as the substrates (from SiMPore, Inc.), which were supported with the amorphous silicon nitride films with thickness of ~5 nm. Before deposition the substrate was heated at 300 °C for 15 min in a high vacuum to desorb volatile contaminants on the surfaces. After cooling the substrate to RT, a 2 Hz KrF laser (wavelength: 248 nm) with about 320 ± 10 mJ cm^−2^ incident fluence was employed for depositing the ultrathin films (thickness <10 nm). Base on the results of X-ray reflectivity and HRTEM for several ultrathin films with different deposition doses (<10 nm), the deposition rate was controlled by the pulse number accurately, and the deposition doses for the samples shown in Fig. 1a, b were 60 and 300 pulses, respectively. To ensure the reliability of testing results, each sample was tested only for one time.

### TEM observations

In this work the in situ HRTEM observations were performed on a Philips CM200 transmission electron microscope operating at 200 kV, and the sample was irradiated with a focused electron beam with an applied continuous current density ≈ 2.1 × 10^6^ electrons nm^−2^ s^−1^ = 33.6 A cm^−2^ (measured from the fluorescent screen), and each frame of image was recorded with a CCD camera with a short exposure time ( ~ 0.5 s). Considering the observational area drifting and sample contamination during testing, the total length of each movie was limited within 30 min.

### Cs-STEM observations

Cs-STEM in situ observations were conducted on a double spherical aberration corrected high resolution scanning transmission electron microscope [JEOL-ARM200F, with cold field-emission gun (CFEG)]. Electrons scattered incoherently from the samples were recorded by a high angle annular dark field (HAADF) detector at an inner collection angle of 62 mrad and convergence angle 19 mrad. A series of images were recorded with a field of view of 16.4 × 16.4 nm^2^, an electron dose of 0.5 × 10^6^ electrons nm^−2^ s^−1^ = 8.5 A cm^−2^, and an acquisition time was 8 s per frame, the time delay between the acquisitions of the two images was 2 s.

### The electron beam irradiation influence

It has been discussed in a lot of the previous works that surface diffusion can be influenced by electron beam irradiation^46,49,55–57^. In the present work, the applied current density was 2.1 × 10^6^ electrons nm^−2^ s^−1^ = 33.6 A cm^−2^ in the HRTEM. In the measurement of the Cs-STEM the probe current was 23 pA, and the equivalent electronic current density was about 0.5 × 10^6^ electrons nm^−2^ s^−1^ = 8.5 A cm^−2^. Compared with the published literatures these values are relatively lower^56–62^. Analyzed by the previous reports, these irradiation doses will increase the temperature of NPs less than 10 K^46–49^. It was reported that the initial metallic NPs (diameters are not larger than 5 nm) are trapped in a deep potential-energy well on the substrate, and a amount of energy is needed to push the NPs out of the large potential well^61^. For the Pd NPs supported by the silicon substrate the range of the initiate electron beam flux is around 10–1000 A cm^−2^ and continuous exposure is in the range of 10-30 s under HRTEM^63^. However, once the initiated NPs start to fluctuate, the energy needed to sustain the state is in much smaller magnitude. Even after turning down the electron beam (almost zero beam intensity), the fluctuating state of the NPs is still sustained^61^, meaning that the liquid-like behavior of NPs is intrinsic^61,64^. From the results of HRTEM, the 200 kV, 33.6 A cm^−2^ continuous electron beam current is enough to decouple the NPs from substrate, and the NPs coalesce subsequently. To minimize the influence of electron beam during the coalescence, we used the atomic resolution Cs-STEM to apply the nano-scale electron beam to scan the image area. As an acquisition time 8 s per frame and 2 s delay time, the total exposure time of a pair of 3 nm diameter NPs in a 16.4 nm × 16.4 nm scanning area is about 0.4 s for each frame at 0.1 Hz frequency under the 200 kV 23 pA electron beam discontinuous irradiation. This short time stimulation is exactly able to activate the NPs into a floating state, and the floating state can persiste for 9.6 s till the next frame scanning. During the whole coalescence process, the NPs endure less than 13.6 μC μm^−2^ electron irradiation dose in a 200 s measurement, and such low irradiation dose even cannot activate the movement of Au NPs from the support^65^. As the result, the estimated viscosity of a-NPs from Cs-STEM is just slightly higher than the value obtained under HRTEM. Therefore, under these conditions the influence of the electron beam irradiation is so modest that it is not a primary factor affecting the liquid-like behavior of the NPs. Even so, it should be indicated that the effect of the energy supply due to the irradiation beam on the viscosity is still under our consideration because a lower irradiation intensity from Cs-STEM produces a slightly higher viscosity as expectable, thus, the determined viscosities are lower limits of the actual viscosity under no irradiation.

## Supplementary information

Supplementary Information

Description of Additional Supplementary Files

Supplementary Movie 1

Supplementary Movie 2

Supplementary Movie 3

Supplementary Movie 4

## Data Availability

The data that support the findings of this study are available from the corresponding author upon reasonable request.
