# Supplementary material for: Liquid-like behaviours of metallic glassy nanoparticles at room temperature
Source: Nat Commun. 2019 Apr 29;10:1966. doi: 10.1038/s41467-019-09895-3 (PMC6488636; doi:10.1038/s41467-019-09895-3)
Supplement: Supplementary file 1 — Supplementary Information [file 41467_2019_9895_MOESM1_ESM.pdf]

Supplementary Information for

Liquid-like Behaviours of Metallic Glassy Nanoparticles at Room Temperature

C. R. Cao[1,2], K. Q. Huang[1,2], J. A. Shi[1,2], D. N. Zheng[1,2], W. H. Wang[1,2, 3], L. Gu[1,2], H. Y. Bai[1,2,3,]*

[1]Institute of Physics, Chinese Academy of Sciences, Beijing 100190, P. R. China

[2]Center of Materials Science and Optoelectronics Engineering, University of Chinese Academy of Sciences, Beijing 100049, China

[3]Songshan Lake Materials Laboratory, Dongguan, Guangdong 523808, China

*To whom correspondence should be addressed. Emails: hybai@iphy.ac.cn

Supplementary Figures

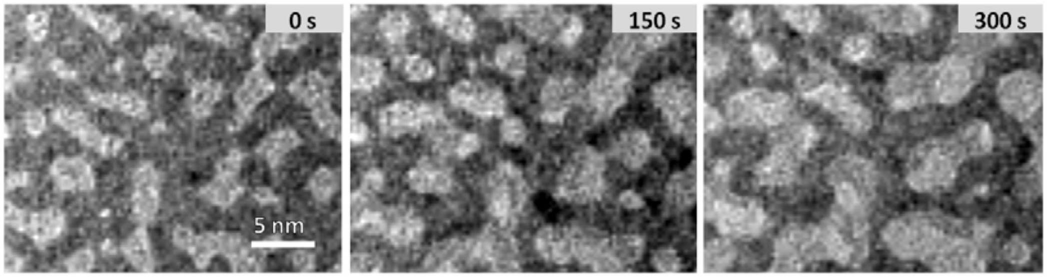

Supplementary Figure 1. A series of images from Supplementary Movie 2 show the relaxation process of the NPs morphology. As the time marked at the top right of images, under the electron beam irradiation the thin film started to shrank and condensed quite slowly, even after 300 s this thicker thin film still shows unapparent shape change.

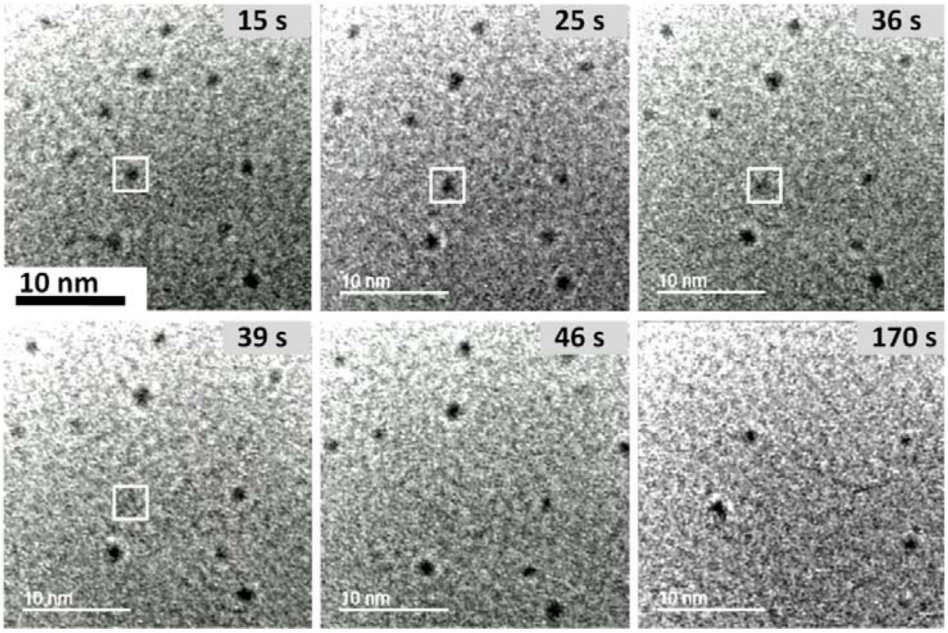

Supplementary Figure 2. A series of images from Supplementary Movie 3 show the Ostwald ripening processes of the small NPs (diameter<1.5 nm). For example, under the continuous irradiation of probe beam the NP marked with a white frame was ripening and gradually disappeared and aggregated to other place at 39 s. Within dozens of seconds the Ostwald ripening processes of such small NPs can be observed clearly before they contact with each other. There are more than 10 NPs in the image labeled 15 s and only four NPs in the image labeled 170 s, indicating that the areal density of NPs decreased significantly during the period of about three minutes in Supplementary Movie 3.
