# Supplementary material for: Liquid-like behaviours of metallic glassy nanoparticles at room temperature
Source: Nat Commun. 2019 Apr 29;10:1966. doi: 10.1038/s41467-019-09895-3 (PMC6488636; doi:10.1038/s41467-019-09895-3)
Supplement: Supplementary file 2 — Description of Additional Supplementary Files [file 41467_2019_9895_MOESM2_ESM.pdf]

Description of Additional Supplementary Files

File Name: Supplementary Movie 1
Description: The ultrathin PdSi film with lower dose (one dose) deposition and composed of the nanoparticles (NPs) with sizes of 1 ~ 3 nm was observed and detected under the detecting electron beam.

File Name: Supplementary Movie 2
Description: The film deposited with higher doses (five doses) compared with the ultrathin film shows sluggish shape change of the PdSi NPs under the same irradiation conditions used in Supplementary Movie 1.

File Name: Supplementary Movie 3
Description: The ultrathin PdSi film deposited with much lower dose (one third dose) and composed of the NPs with sizes of 1~1.8 nm shows the remarkable Oswald ripening process in which the NPs diffuse rapidly under the same irradiation conditions used in Supplementary Movie 1.

File Name: Supplementary Movie 4
Description: The PdSi film deposited with mild moderate dose (three doses) and composed of the NPs with sizes of 2~6 nm was exposed under the same irradiation conditions used in Supplementary Movie 1 for 10 minutes.
